# Prognostic factors in patients with localized and metastatic alveolar rhabdomyosarcoma. A report from two studies and two registries of the Cooperative Weichteilsarkom Studiengruppe CWS

**DOI:** 10.1002/cam4.70215

**Published:** 2025-01-09

**Authors:** Ewa Koscielniak, Sabine Stegmaier, Gustaf Ljungman, Bernarda Kazanowska, Felix Niggli, Ruth Ladenstein, Bernd Blank, Erika Hallmen, Christian Vokuhl, Claudia Blattmann, Monika Sparber‐Sauer, Thomas Klingebiel

**Affiliations:** ^1^ Olgahospital, Pediatrics 5 (Oncology, Hematology, Immunology) Klinikum Stuttgart Stuttgart Germany; ^2^ Medical Faculty University of Tübingen Tübingen Germany; ^3^ Department of Women's and Children's Health, Pediatric Oncology Uppsala University Uppsala Sweden; ^4^ Department of Pediatric Hematology/Oncology and BMT University of Wroclaw Wroclaw Poland; ^5^ Department of Pediatric Oncology University of Zürich Zurich Switzerland; ^6^ Department of Studies and Statistics for Integrated Research and Projects and Medical University of Vienna, Paediatric Department St. Anna Children's Hospital, Children's Cancer Research Institute Vienna Austria; ^7^ Department of Pathology, Section of Pediatric Pathology University Bonn Bonn Germany; ^8^ Department for Children and Adolescents University Hospital Frankfurt, Goethe University Frankfurt am Main Germany

**Keywords:** alveolar, *FOXO1‐*fusion, *PAX3* *::* *FOXO1* fusion, prognostic factors, rhabdomyosarcoma, risk stratification

## Abstract

**Background:**

The histologic classification of rhabdomyosarcoma (RMS) as alveolar (aRMS) or embryonal (eRMS) is of prognostic importance, with the aRMS being associated with a worse outcome. Specific gene fusions (*PAX3/7* *::* *FOXO1*) found in the majority of aRMS have been recognized as markers associated with poor prognosis and are included in current risk stratification instead of histologic subtypes in localized disease. In metastatic disease, the independent prognostic significance of fusion status has not been definitively established. The objective of this analysis was to evaluate survival outcomes of patients with localized and metastatic aRMS and its association with fusion status and subtype (*PAX3/7* *::* *FOXO1*, *FOXO1* break), and clinical prognostic factors.

**Methods:**

A total of 470 patients with aRMS ≤21 years of age enrolled in two CWS‐trials and two registries was eligible for the analysis.

**Results:**

The 5‐year event‐free survival (EFS) and overall survival (OS) rates for all patients with localized vs. metastatic tumors were: 56% and 65% vs. 18% and 22%, respectively. Of the 368 (78%) tumors tested, specific fusion was found in 330 (90%), considered “fusion positive” FP (*PAX3* *::* *FOXO1* in 280, *PAX7* *::* *FOXO1* in 49, *FOXO1* break in 59 tumors). In patients with localized tumors, univariate analysis revealed that clinical group, tumor invasiveness (T1 vs.T2), regional lymph node involvement (N0 vs. N1) and *FOXO1* fusion were significantly associated with EFS and OS, tumor size and *PAX* variant with OS only. In patients with metastatic aRMS, age, bone/marrow (B/BM) metastases, *FOXO1* fusion and *PAX* variant were associated with EFS and OS, T status with OS only. Multivariate analysis identified *PAX3* *::* *FOXO1* fusion as an independent adverse prognostic factor for EFS in patients with localized disease and for EFS and OS in patients with metastatic disease, B/BM metastases for EFS.

**Conclusion:**

*PAX3* *::* *FOXO1* fusion should replace *FOXO1* fusion as an adverse prognostic factor in risk stratification. The prognostic relevance of *PAX7* *::* *FOXO1*‐positive and *FOXO1* fusion negative aRMS, along with the clinical factors described in this report, allows further refinement of risk assessment of patients with localized and metastatic aRMS.

## Graphical abstract

*PAX3::FOXO1* fusion should replace *FOXO1* fusion as an adverse prognostic factor in risk stratification of patients with rhabdomyosarcoma. The prognostic relevance of *PAX7::FOXO1*‐positive and FN alveolar rhabdomyosarcoma (aRMS), along with the clinical factors described in this report, allows further refinement of risk assessment of patients with localized and metastatic (aRMS).

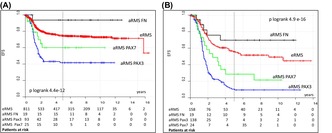

## 1 INTRODUCTION

Rhabdomyosarcomas (RMS) are a heterogeneous group of malignancies of mesenchymal cell origin and are the most common soft tissue sarcoma (STS) of childhood accounting for more than 50% of all cases in patients aged <18 (kinderkrebsregister.de).

The cure rate of children with localized RMS has increased from 25% in 1970 to over 70% in 1990.^1^, ^2^ In the last three decades, however no further significant improvements in survival rates have been achieved but the cumulative therapy burden has become better adapted to risk profile allowing for minimization of severe late effects.^3^, ^4^ An exception is the RMS 2005 study of the European pediatric Soft Tissue Sarcoma Study Group (EpSSG), which showed a 13% improvement in OS with maintenance therapy in patients with high‐risk RMS.^5^ In patients with metastatic disease outcome has not improved significantly and remains poor.^6^, ^7^ Factors defined as directly influencing prognosis, such as patient age, histologic subtype, tumor site, size, invasiveness, resectability and regional lymph node involvement have been incorporated into risk‐adapted stratification systems for patients with localized RMS.^4^, ^8^, ^9^ In metastatic disease, factors associated with prognosis have been defined but have not been widely used to stratify therapy.^10^, ^11^ In the pediatric population two major RMS subtypes exist: “alveolar” (aRMS) and “embryonal” (eRMS), whereas tumors with pleomorphic and spindle cell/sclerosing cell histology account for the majority of RMS diagnosed in individuals >18 years of age. Chromosomal translocations resulting in fusion of the DNA‐binding domain of the *PAX3* or *PAX7* genes to the transactivation domain of the *FOXO1* gene have been detected in approximately 80% of aRMS and a small subset express rare variants such as *PAX3* *::* *FOXO4* or *PAX3*::*NCOA1/2*.^12^, ^13^, ^14^, ^15^ aRMS expressing either *PAX* *::* *FOXO1* gene share a common expression profile distinct from fusion‐negative aRMS and from other RMS variants.^16^, ^17^ aRMS subtype was known as being strongly associated with poor prognosis and was regarded in the past as a “unfavorable” histology in risk stratification for patients with localized tumors.^3^, ^4^, ^18^ However, histologic subtype was not an independent prognostic factor in an analysis of patients with metastatic disease treated in nine trials conducted by European and American cooperative groups.^11^ Several studies over the past 20 years have reported that the presence of the *FOXO1*‐fusion, rather than alveolar histology, is associated with poor outcome.^17^, ^19^, ^20^, ^21^ *FOXO1* status has been shown to be the most important prognostic factor in patients with RMS after metastatic status in an analysis of six Children's Oncology Group (COG) clinical trials and is included in risk stratification for patients with localized disease of the ongoing COG and EpSSG trials (COG ARST1431 NCT02567435 and EpSSG FaR‐RMS NCT 04625907).^22^ In metastatic disease, the independent prognostic significance of fusion status has not been definitively established.^6^, ^23^ However, most analyses evaluating clinical factors influencing outcome have included patients with all histologically or molecularly defined subtypes of RMS.^9^, ^22^

In the recently published European studies, all patients with localized aRMS were included in the high and very high (patients with lymph node involvement) risk groups, regardless of other known clinical risk factors.^3^, ^4^ Similarly, patients with *FOXO1* fusion‐positive localized RMS without lymph node involvement will not be further stratified in the ongoing EpSSG and COG trials.^9^ More recently, an analysis of patients with localized *FOXO1* fusion‐positive RMS treated in three COG trials has shown that this group can be further stratified based on clinical characteristics.^24^ However, it has not yet been investigated which clinical factors, in addition to fusion status and subtype, influence the prognosis of patients with aRMS. The purpose of this analysis was therefore to evaluate survival outcomes of patients with localized and metastatic aRMS and its association with fusion status (*PAX3/7* *::* *FOXO1*, *FOXO1* break) and type (*PAX3*‐ vs. *PAX7* *::* *FOXO1*) and clinical prognostic factors in patients enrolled in two studies: CWS‐2002P, CWS‐IV 2002, and two registries: CWS‐DOK IV 2004 and the European Soft Tissue Sarcoma Registry SoTiSaR.

## 2 MATERIALS AND METHODS

**FIGURE 1:**
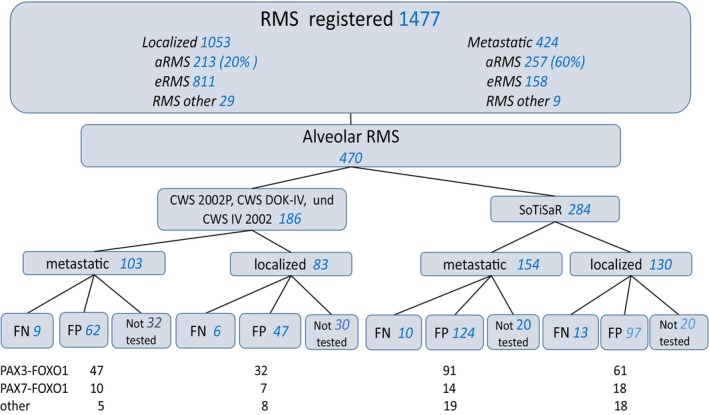
Flowchart of patients included in the analysis.

Patients with aRMS ≤21 years of age with sufficient clinical data registered in two trials (CWS‐2002P 17.1.2003‐31.12.2010, CWS‐IV 2002 10.1.2005‐31.12.2007) and two registries (CWS‐DOK IV 10.1.2005‐31.12.2010, SoTiSaR 1.7.2009‐31.12.2017) were eligible for the analysis. Patients with localized eRMS from the same trials were included in the survival analysis only as a reference group (Figure 1). CWS‐2002P was designed as prospective, non‐randomized, historically controlled trial on localized RMS and non‐rhabdomyosarcoma soft tissue sarcoma (NRSTS).^4^ CWS‐IV 2002 was an open Phase II window study and CWS‐DOK IV 2004 was a prospective registry for patients aged ≤21 years with metastatic soft tissue sarcoma (STS).^25^, ^26^ SoTiSaR is a prospective European Registry for STS in patients aged ≤21 years. Participating countries are Austria, Germany, Poland, Sweden, Switzerland and Finland. Therapy details of CWS‐2002P and CWS‐IV 2002, CWS‐DOK‐IV 2004 and CWS‐Guidance (treatment recommendations for patients enrolled on SoTiSaR) have already been published.^4^, ^6^, ^25^, ^26^, ^27^ Briefly: patients with localized aRMS were assigned to the high and very high‐risk group. Risk stratification is shown in Table S1. Patients with metastatic disease were not further stratified. All patients received multimodal treatment with chemotherapy, surgery and/or radiotherapy according to the respective protocol, patients enrolled in the SoTiSaR according to the CWS‐Guidance. Chemotherapy consisted of three or four drugs (ifosfamide, vincristine, actinomycin and doxorubicin). In patients with metastatic tumors, doxorubicin was replaced by eprirubicin and etoposide and carboplatin were added. More information on the details of therapy is available in the Supporting information (Methods, Figures S1 and S2). Informed consent was obtained from all parents/guardians or patients according to the legal requirements and the Declaration of Helsinki. CWS‐2002P, CWS‐IV 2002 and SoTiSaR were approved by Ethics Review Board of the University of Tübingen (51/2003, 218/2000 and 158/2009B02). Ethical review and approval were waived for CWS‐DOK IV 2004, as it was considered an addendum to the CWS‐2002P. All diagnoses were confirmed by central pathology review (C.V).In addition to the typical morphology of aRMS, P‐cadherin positivity, with the absence of EGFR staining and documentation of a sparse reticulin fiber network by reticulin staining, was required to make a diagnosis of aRMS.^12^ Molecular testing was used to detect *PAX3/7* *::* *FOXO1* or *PAX3* *::* *NCOA1/2* by RT‐PCR and/or FISH (*FOXO1* break) and was performed centrally (S.S. and C.V) as previously described.^12^, ^28^, ^29^, ^30^, ^31^ aRMS negative in both tests were described as “fusion negative” FN aRMS. If the testing was not performed the tumor was diagnosed based on morphological criteria only. C.V conducted a histology re‐review of all cases classified as alveolar but lacking fusion transcripts. In 8 FN aRMS cases, tumor samples were available for RNA sarcoma panel sequencing, which did not reveal any other rare fusions. Rare fusions known to occur in aRMS were included in the panel. Details on genes of the panel are available in the Data S1.

### 2.1 Statistical methods

Statistical analyses were performed using SPSS statistics 22–25.0.0 (IBM Corporation. Armonk. NY) and R 3.02 (Bell Laboratories. Murray Hill. NJ. USA) software packages. Continuous variables were summarized by indicating their interquartile ranges whereas categorical variables were reported as counts and percentages. Clinical variables and its frequency distributions were compared by the χ^2^‐test or by the Fisher‐test. Event free survival (EFS) and overall survival (OS) were calculated using Kaplan–Meier method. EFS was calculated as the time elapsed between the date of diagnosis and either the occurrence of an event or the date of the last patient contact. Event was defined as relapse of disease (local, metastatic or combined) in patients who achieved complete remission, disease progression (defined as growth of tumor in patients who did not achieved complete remission) or death. Second malignancy was not defined as an event as described in the protocols. OS was defined as time from diagnosis to death or last follow up for surviving patients. Patients who had not experienced an event at their last contact were considered censored. Difference in outcome rates (survival curves) were estimated by the log‐rank test statistic (Mantel–Haenszel Test) at an alpha level of 0.05 (5%). Multivariate analysis was performed using Cox's proportional hazards to simultaneously assess the effect of the variables tested in the univariate analysis, with the exception of tumor site, the inclusion of which resulted in numerical instability “loklik converged before variabe 3,4 coefficient may be infinite”. Fusion‐positive tumors could not be analyzed as a whole group in the multivariate analysis when evaluated in strata due to their correlation (to avoid multicollinearity). The proportional hazards assumption was tested by checking the Schoenfeld residuals.

## 3 RESULTS

Eligibility criteria were fulfilled in 470 patients (213 with localized and 257 with metastatic disease). The slightly higher number of patients with metastatic versus localized disease reflects the known much higher incidence of the alveolar subtype in metastatic RMS.

The distribution of patients by study and disease extent is shown in Figure 1. Patient characteristics are presented in Table 1.

**TABLE 1:** Patient characteristics.

Variable	Localized		Metastatic		Total	
	213	%	257	%	470	%
Gender						
Male	104	48.8	126	49.0	230	48.9
Female	108	50.7	130	50.6	238	50.6
Missing data	1	0.5	1	0.4	2	0.4
Age						
≤10 years	126	59.2	98	38.1	224	47.7
>10 years	87	40.8	159	61.9	246	52.3
Tumor size						
≤5 cm	106	49.8	61	23.7	167	35.5
>5 cm	91	42.7	165	64.2	256	54.5
Missing data	16	7.5	31	12.1	47	10.0
Tumor site						
EXT	68	31.9	119	46.3	187	39.8
HN‐nPM	39	18.3	3	1.2	42	8.9
HN‐PM	55	25.8	31	12.1	86	18.3
ORBITA	8	3.8	0	0.0	8	1.7
GU‐BP	5	2.3	13	5.1	18	3.8
GU‐nBP	7	3.3	5	1.9	12	2.6
Other	31	14.6	79	30.7	110	23.4
Missing data	0	0.0	7	2.7	7	1.5
Tumor Site Risk group						
Favorable (ORB, GU‐nBP, HN‐nPM)^a^	54	25.3	8	3.1	62	13.2
Unfavorable (EXT, HN‐PM, GU‐BP, Other)^b^	159	74.7	242	94.2	401	85.3
Missing data	0	0	7	2.7	7	1.5
IRS Group						
I	10	4.7	0	0.0	10	2.1
II	29	13.6	0	0.0	29	6.2
III	174	81.7	0	0.0	174	37.0
IV	0	0.0	257	100.0	257	54.7
Risk group						
High	136	63.8	0	0.0	136	28.9
Very high	75	35.2	0	0.0	75	16.0
Metastatic	0	0.0	257	100.0	257	54.7
Missing data	2	0.9	0	0.0	2	0.4
T‐status						
T1	99	46.5	34	13.2	133	28.3
T2	95	44.6	205	79.8	300	63.8
Tx (missing data)	19	8.9	18	7.0	37	7.9
N‐status						
N0	126	59.2	53	20.6	179	38.1
N1	75	35.2	160	62.3	235	50.0
NX (missing data)	12	5.6	44	17.1	56	12.0
B/BM						
Yes			158	61.5		
No			93	36.2		
Missing data			6	2.3		
Study						
SoTisaR	130	61.0	154	59.9	284	60.4
CWS‐2002P, CWS‐DOK IV CWS‐IV 2004	83	39.0	103	40.1	186	39.6
*FOXO1* fusion status						
Negative	19	8.9	19	7.4	38	8.1
Positive	144	67.6	186	72.4	330	70.2
Not known	50	23.5	52	20.2	102	21.7

^a^ ORB, orbita; GU‐nBP, genitourinary‐non bladder/prostate; HN‐nPM, head/neck‐non parameningeal.

^b^ EXT, extremity; HN‐PM, head/neck‐parameningeal; GU‐BP, genitourinary‐bladder/prostate.

Median follow‐up for all patients was 8.3 years (IQR 5.3–10.7). The estimated 5‐year EFS and OS rates for all patients with localized tumors were: 56% (95% CI 49–63) and 65% (95% CI 58–72), for patients with metastatic disease 18% (95% CI 14–24) and 22% (95% CI 17–28) respectively. There was no difference in EFS and OS between the different CWS cohorts (Tables 2 and 3). 368 tumors (78%) were tested by FISH and/or RT‐PCR, of which the specific fusion was found in 330 (90%) aRMS (“fusion positive” FP). 38 tumors (10%) were classified as “fusion negative” FN aRMS. In 102 (22%) tumors the fusion status was not known. The *PAX* variant was available for 280 (85%) FP tumors, with *PAX3* *::* *FOXO1* representing the vast majority (*n* = 231, 82%). *PAX7* *::* *FOXO1* was found in 49 tumors (18%). In 50 the *FOXO1* fusion partner was not known. No *PAX3* *::* *NCOA1/2* fusion was found in the cohort studied. There was no difference in the distribution of patient and tumor‐related variables between patients with FP and FN localized aRMS, in contrast to patients with metastatic tumors, where age >10 years, unfavorable primary sites and bone and/or bone marrow (B/BM) involvement were significantly more frequent in the FP group (Tables S2 and S3).

**TABLE 2:** Univariate analysis for EFS and OS. Patients with localized aRMS.

Variable	*n*	%	5 year EFS % (95% CI)	*p* logrank	5 year OS % (95% CI)	*p* logrank
Study						
SoTiSaR	130	61.0	54.6 (45.9–65.0)	0.39	65.2 (56.1–75.7)	0.95
CWS‐2002P	83	39.0	58.7 (48.8–70.6)		63.9 (54.0–75.6)	
Age						
≤10	126	59.2	57.7 (49.0–67.9)	0.37	65.1 (56.3–75.4)	0.46
>10	87	40.8	53.6 (43.6–65.9)		64.1 (54.0–76.0)	
Tumor size						
≤5 cm	106	49.8	60.9 (51.7–71.8)	0.24	70.9 (61.7–81.4)	0.04
>5 cm	91	42.7	52.1 (42.3–64.2)		60.4 (50.3–72.4)	
Missing data	16	7.5				
Tumor site						
Favorable^a^	54	25.4	57.2 (44.7–73.2)	0.95	70.1 (57.6–85.5)	0.23
Unfavorable^b^	159	74.6	55.7 (48.0–64.6)		62.8 (54.9–71.8)	
IRS‐group						
I	10	4.7	76.2 (52.1–100.0)	0.05	80.0 (51.6–100.0)	0.05
II	19	8.9	76.3 (61.4–94.9)		83.1 (69.2–99.8)	
III	174	81.7	51.8 (44.4–60.4)		60.7 (53.1–69.4)	
T‐status						
T1	99	46.5	66.0 (56.7–76.9)	0.07	75.3 (66.1–85.7)	0.003
T2	95	44.6	46.6 (37.2–58.4)		54.1 (44.2–66.3)	
TX	19	8.9				
N‐status						
N0	126	59.2	50.2 (42.9–58.8)	0.002	56.4 (48.8–65.2)	7.0e‐04
N1	75	35.2	28.1 (22.6–35.0)		33.1 (27.1–40.5)	
NX	12	5.6				
Fusion status						
Positive	144	67.6	47.8 (39.9–57.4)	0.002^c^	59.5 (51.0–69.4)	0.009^c^
Negative	19	8.9	93.7 (82.6–100.0)		93.7 (82.6–100.0)	
Not known	50	23.5	−63.3 (49.7–80.5)		71.4 (8.1–87.8)	
Pax variant						
*PAX3* *::* *FOXO1*	93	43.7	42.0 (32.8–53.8)	0.15	50.5 (40.4–63.1)	0.03^d^
*PAX7* *::* *FOXO1*	25	11.7	60.4 (43.1–84.7)		80.9 (65.6–99.7)	
*PAX* not known	26	12.2	66.0 (48.1–90.5)		68.4 (50.1–93.3)	

^a^ ORB, orbita; GU‐nBP, genitourinary‐non bladder/prostate; HN‐nPM, head/neck‐non parameningeal.

^b^ EXT, extremity; HN‐PM, head/neck‐parameningeal; GU‐BP, genitourinary‐bladder/prostate.

^c^ p for positive versus negative.

^d^ p for *PAX3* versus *PAX7*.

**TABLE 3:** Univariate analysis for EFS and OS. Patients with metastatic aRMS.

	*n* (257)	%	5 year EFS % (95% CI)	*p* logrank	5 year OS % (95% CI)	*p* logrank
Study						
SoTiSaR	154	59.9	18.1 (12.6–26.2)	0.79	21.7 (15.4–30.6)	0.83
CWS‐IV‐2002 CWS‐DOK IV	103	40.1	17.9 (11.7–27.4)		21.3 (14.6–31.3)	
Age						
≤10	98	38.1	32.1 (23.5–43.9)	6.3 e‐04	39.0 (29.7–51.3)	6.8 e‐05
>10	159	61.9	9.9 (6.1–16.2)		11.6 (7.2–18.6)	
Gender						
Male	130	50.6	21.2 (14.9–30.2)	0.30	22.0 (15.4–31.5)	0.99
Female	126	49.0	15.4 (10.0–23.7)		21.4 (14.9–30.7)	
Missing data	1	0.4				
Tumor size						
≤5 cm	61	23.7	18.1 (10.4–31.6)	0.41	21.8 (13.0–21.6)	0.45
>5 cm	165	64.2	20.3 (14.7–27.9)		24.3 (18.2–32.5)	
Missing data	31	12.0				
Tumor site						
Favorable^a^	8	3.1	29.2 (9.1–93.2)	0.30	33.3 (10.8–100.0)	0.20
Unfavorable^b^	242	94.2	17.9 (13.4–23.8)		21.7 (16.7–28.1)	
Missing data	7	2.7				
T‐status						
T1	34	13.2	25.3 (13.9–46.0)	0.20	44.1 (29.4–66.2)	0.05
T2	205	79.8	18.1 (13.2–24.7)		19.8 (14.6–26.7)	
Missing data	18	7.0				
N‐status						
N0	53	20.6	11.4 (4.9–26.5)	0.40	15.0 (7.3–30.7)	0.40
N1	160	62.3	20.6 (14.9–28.4)		24.1 (17.8–32.5)	
Missing data	44	17.0				
B/BM^c^ metastases						
Yes	159	61.9	11.4 (7.2–18.1)	6.0e‐05	12.2 (7.8–19.1)	4.0e‐05
No	91	35.4	30.3 (21.6–42.4)		37.6 (28.0–50.0)	
Missing data	8	2.7				
Fusion status						
Positive	186	72.4	10.8 (7.0–16.7)	6.0 e‐06^d^	14.8 (10.2–21.5)	7.0e‐05^d^
Negative	19	7.4	71.0 (52.3–96.4)		70.1 (51.0–96.1)	
Not known	52	20.2	27.5 (17.0–44.5)		30.1 (18.8–48.5)	
Fusion variant						
*PAX 3* *::* *FOXO1*	138	53.7	7.7 (4.2–14.0)	0.04	9.8 (5.6–17.1)	0.03^e^
*PAX 7* *::* *FOXO1*	24	9.3	17.2 (6.6–44.7)		28.3 (14.1–56.9)	
PAX not known	24	9.3	25.2 (12.1–52.4)		29.3 (15.0–57.2)	

^a^ ORB, orbita; GU‐nBP, genitourinary‐non bladder/prostate; HN‐nPM, head/neck‐non parameningeal.

^b^ EXT, extremity; HN‐PM, head/neck‐parameningeal; GU‐BP, genitourinary‐bladder/prostate.

^c^ B/BM, bone/bone marrow.

^d^ p for positive versus negative.

^e^ p for PAX3 versus PAX7.

When comparing clinical characteristics between *PAX3* *::* vs. *PAX7* *::* *FOXO1* positive localized aRMS, the only difference was a higher percentage of non‐invasive tumors (“T1”) in *PAX7* *::* *FOXO1* group (Table S4). In metastatic disease, patients with *PAX7* *::* *FOXO1* aRMS were significantly younger compared to *PAX3* *::* *FOXO1* positive group (68% vs. 33% of patients ≤10 years of age), all other factors were similarly distributed (Table S5).

On univariate analysis in patients with localized disease, IRS (Intergroup Rhabdomyosarcoma Study) group, regional lymph node involvement (N0 vs.N1) and *FOXO1* fusion status were associated with EFS and OS (Table 2). Patients with IRS group III, N1, and FP tumors had significantly inferior EFS and OS compared to those with IRS group I‐II, N0 status and FN aRMS. For tumor size, tumor invasiveness (T1 vs. T2) and *FOXO1* fusion partner (*PAX3* vs. *PAX7*) a significant correlation was observed for OS (Table 2). Patients with invasive (T2), large (>5 cm) tumors and bearing *PAX3* *::* *FOXO1* fusion had significantly inferior OS compared to those with non‐invasive (T1), smaller ≤5 cm and *PAX7* *::* *FOXO1* positive tumors. In multivariate analysis, only *PAX3* *::* *FOXO1* fusion status emerged as independent prognostic factor for EFS (Table 4).

**TABLE 4:** Multivariate analysis for EFS and OS in patients with localized and metastatic aRMS.

	Localized^a^							Metastatic^b^						
	*n*	HR EFS	95% CI	*p*‐value	HR OS	95% CI	*p*‐value	*n*	HR EFS	95% CI	*p*‐value	HR OS	95% CI	*p*‐value
Age														
≤10 years	79	1.0			1.0			58	1.0			1.0		
>10 years	52	1.21	0.67–2.2	0.52	1.15	0.58–2.97	0.68	86	0.99	0.63–1.56	0.99	1.09	0.67–1.77	0.73
Tumor size														
≤5 cm	66	1.0			1.0			35	1.0			1.0		
>5 cm	65	1.45	0.49–1.74	0.80	1.06	0.51–2.17	0.89	109	0.71	0.44–1.15	0.17	0.74	0.45–1.20	0.22
IRS														
I + II	21	1.0			1.0									
III	110	1.44	0.53–3.96	0.47	1.60	0.45–5.73	0.47							
T‐status														
T1	68	1.0			1.0			16	1.0			1.0		
T2	63	1.18	0.62–2.25	0.62	1.22	0.58–2.56	0.60	128	1.22	0.61–2.45	0.57	1.82	0.83–4.01	0.14
N‐status														
N0	83	1.0			1.0			13	1.0			1.0		
N1	48	1.43	0.76–2.67	0.26	1.60	0.78–3.27	0.20	111	0.77	0.49–1.20	0.25	0.87	0.55–1.40	0.57
B/BM														
Yes								93	1.0			1.0		
No								51	0.61	0.39–0.97	0.04	0.66	0.41–1.09	0.09
Fusion														
Negative	17	1.0			1.0			13	1.0			1.0		
*PAX3 pos*	74	9.34	1.29–69.1	0.03	6.52	0.88–48.5	0.07	95	5.33	1.89–15.1	0.002	4.41	1.57–12.5	0.005
*PAX7 pos*	22	7.29	0.89–59.5	0.06	4.19	0.49–36.0	0.19	16	2.67	0.85–8.36	0.09	2.10	0.65–6.81	0.21
*PAX* not known	18	3.58	0.37–34.7	0.27	2.34	0.21–26.1	0.49	20	3.09	0.86–8.36	0.06	2.59	0.79–8.53	0.12

*Note: PAX3* vs.−*PAX7* HR EFS *p* = 0.55, HR OS *p* = 0.37 and *PAX3* vs.‐ *PAX7* HR EFS 0.04, pHR OS 0.04.

^a^ Number of events for EFS 50, for OS 37.

^b^ Number of events for EFS 113, for OS 104.

Among patients with metastatic disease, patients aged >10 years, with FP tumors, *PAX3* *::* *FOXO1* fusion type and B/BM involvement, had significantly worse EFS and OS compared to patients aged ≤10 years, without B/BM involvement, FN tumors, or with *PAX7* *::* *FOXO1* fusion type (Table 3). Tumor invasiveness (T2) was adversely associated with OS only. Multivariate analysis in patients with metastatic tumors, identified *PAX3* *::* *FOXO1* fusion as an independent adverse prognostic factor for EFS and OS, B/BM involvement for EFS only (Table 4).

**FIGURE 2:**
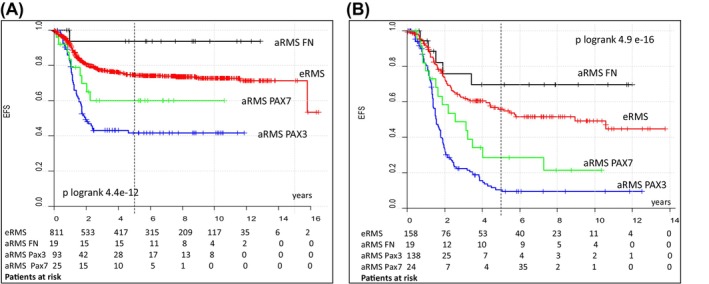
EFS in patients with localized (A) and metastatic (B) tumors according to histology, fusion status and fusion type.

When comparing the outcomes of patients with FN‐, *PAX3* *::* or *PAX7* *::* *FOXO1*‐positive localized aRMS to those with eRMS treated in the CWS trials included in this analysis (Figure 2, Table S6), patients with *PAX3* *::* *FOXO1*‐positive tumors had significantly worse EFS and OS. Patients with FN aRMS had better EFS and OS without reaching statistical significance (5 year EFS 94% vs. 74%, 5 year OS 94%vs. 83%) and the results for patients with *PAX7* *::* *FOXO1*‐positive tumors were comparable to those for eRMS (5 year EFS 60% vs. 74%, 5 year OS 80%vs. 83%). In patients with metastatic tumors, *PAX3* *::* *FOXO1* and *PAX7* *::* *FOXO1*‐positive aRMS had significantly worse EFS and OS compared to patients with eRMS in contrast to patients with FN aRMS whose EFS and OS were much better but did not reach statistical significance (5 year EFS 71% vs. 45%, 5 year OS 70%vs. 56%).

## 4 DISCUSSION

The present analysis describes the survival outcomes in a large group of patients with localized and metastatic aRMS, prospectively enrolled and uniformly treated in recent CWS trials and registry, in relation to *FOXO1* fusion status and fusion type (*PAX3* vs. *PAX7*) in the context of known clinical factors associated with prognosis. The percentage of FN aRMS in our analysis was lower (10%) than in other published series which reported 16%–38%, probably due to the differences in the diagnostic criteria for aRMS.^12^, ^20^, ^21^, ^23^, ^32^, ^33^ The International Classification of RMS (ICR) published in 1995 modified the diagnostic criteria for aRMS.^34^ This change resulted in an increase in the frequency of aRMS and doubled the proportion of *FOXO1* fusion negative aRMS cases to 37% on D9803.^23^ Upon re‐review using current diagnostic criteria the percentage of FN aRMS decreased to 18%. The proportion of FN aRMS in the analysis of patients treated in MMT trials in the UK was also much higher at 20/54 (38%).^32^ All of these differences and changes in diagnostic criteria may affect not only the incidence of aRMS, but likely also the incidence, composition, and prognosis of FN aRMS. In the CWS studies, which included immunohistomorphology early in their classification system, the introduction of ICR did not result in major changes and the proportion of aRMS remains constant at around 15%–20% with FN aRMS of 6%–11%.^2^, ^4^, ^6^, ^35^ The *FOXO1* fusion negative aRMS has been described as a molecularly heterogeneous group comprising cases with rare fusion‐positive cells, cases with alternate rare genetic fusions, cases with genomic fusions and no detectable RNA product and cases with no evidence of molecular fusion.^30^, ^36^

Because patients with localized and metastatic RMS have different distributions of histologic subtypes, clinical characteristics and prognosis, we analyzed and discussed these two groups separately.

Interestingly, we found no differences in the distribution of clinical features between FP‐ and FN‐localized aRMS. When comparing localized *PAX3* *::* vs. *PAX7* *::* *FOXO1* positive tumors, the only difference was the significantly higher percentage of less invasive (T1) tumors in the *PAX7* *::* *FOXO1* positive group. Williamson et al. reported that the proportion of tumors occurring in favorable sites is significantly lower in FP than in FN aRMS, but patients with localized and metastatic disease were analyzed together.^20^ An association between a higher rate of *PAX7* *::* *FOXO1* fusion and low‐risk clinical features was found in the analyses of COG D9602 and D9803.^21^, ^37^ Differences in the composition of the cohorts studied may limit comparability with our results. Despite similarities in the distribution of clinical factors between patients with FP and FN localized tumors in our cohort, fusion status together with IRS group and lymph node status were significantly associated with EFS and OS on univariate analysis. Tumor size, invasiveness, and *PAX* variant were predictive for OS. Patients with *PAX7* *::* *FOXO1* positive tumors had a significantly better OS compared to those with *PAX3* *::* *FOXO1* positive tumors (81% vs. 50%, *p* = 0.03), slightly worse compared to FN (81% vs. 94%) and similar to those with eRMS (81% vs. 84%). Multivariate analysis showed that only *PAX3* *::* *FOXO1* fusion was an independent adverse prognostic factor for EFS in patients with localized tumors.

Many studies have identified *FOXO1* fusion status as a prognostic factor in RMS and have also reported on the additional impact of clinical factors, but most have analyzed localized and metastatic tumors together and been conducted with convenience cohorts.^16^, ^19^, ^20^, ^38^, ^39^ Few have analyzed the impact of fusion status and other prognostic factors in patients with localized RMS treated in prospective trials and differentiated between FN eRMS and FN aRMS.^21^, ^22^, ^24^, ^33^, ^37^, ^40^ They found that the prognostic significance of fusion status and its type depends on other clinical factors and study cohorts. In the COG and EpSSG analysis of patients with aRMS and lymph node involvement (N1), EFS for patients with *FOXO1*‐positive tumors treated on EpSSG RMS 2005 was significantly inferior to those *FOXO1*‐negative. Tumor invasiveness (T) was identified as an additional independent prognostic factor. In contrast, in the COG trial ARST0531, the EFS of patients with *FOXO1‐*positive and negative N1 aRMS was similar.^40^

An analysis of the COG D9803 trial showed that OS was significantly inferior for patients with *PAX3* *::* *FOXO1* positive RMS compared to the *PAX7* *::* *FOXO1*positive group, whose OS was comparable to that of patients with FN aRMS and eRMS, in line with our results. However, the significance of *PAX* variant for OS decreases when analyzed in patients with favorable stage and IRS group.^21^

In the analysis of patients with FP localized aRMS treated on three COG studies (D9602, D9803 and ARST0531) an association with EFS was found for patient age, tumor size and invasiveness. Age, IRS group, tumor size, site and invasiveness, nodal status and fusion type (*PAX7* *::* vs. *PAX3* *::* *FOXO1*) were significantly associated with OS.^24^ It is intriguing that in our and COG analysis there was a large overlap in clinical factors associated with OS (IRS group, tumor size and invasiveness, nodal status and fusion type *PAX3* *::* vs. *PAX7* *::* *FOXO1)* but different factors were associated with EFS (COG: age, tumor size and invasiveness, CWS: IRS group and nodal status). Surprisingly, in the COG analysis lymph node involvement (N0 vs. N1) had no impact on EFS (4‐year EFS 56% vs. 47%). The authors suggested that tumor size may contribute more than lymph node involvement in predicting the likelihood of relapse in patients with localized FP RMS. Multivariate analysis of the COG cohort identified older age (≥10 years) and tumor size >5 cm as independent adverse prognostic factors for EFS, and for OS in addition to tumor size, tumor invasiveness (T2) and *PAX3* *::* *FOXO1* variant. The correlation of *PAX3* *::* *FOXO1* with inferior OS decreased; however, when associated with other adverse prognostic factors such as large tumor size and older age.^24^ Despite differences in the prognostic significance of *PAX3* *::* *FOXO1* versus *PAX7* *::* *FOXO1* fusion for EFS and OS between the two COG analyses and ours, there is a consensus that *PAX3* *::* *FOXO1* fusion is associated with poor prognosis rather than *PAX7* *::* *FOXO1*. The prognostic significance of *PAX* variants has also been previously reported based on convenience cohorts.^19^, ^41^, ^42^ However, differences in prognosis between patients with *PAX7* and *PAX3* fusion positive tumors have not been recognized as sufficiently proven to be incorporated into current risk stratification. Many molecular differences that support the clinical differences have been reported. *PAX7* *::* *FOXO1* fusion gene is usually amplified while the *PAX3* *::* *FOXO1* fusion gene is rarely amplified.^43^

It has been shown that the *PAX3* *::* *FOXO1*‐ and *PAX7* *::* *FOXO1* positive subsets are molecularly distinguished by DNA methylation suggesting that epigenetic differences may contribute to the distinct biology and clinical features between these two FP subsets.^44^

Manceau et al. showed that *PAX3* *::* *FOXO1* and *PAX7* *::* *FOXO1* generate partially divergent transcriptomic signatures, which include genes encoding regulators of cell morphology and cell cycle and demonstrated a differential mode of action between the two chimeric proteins that could in turn support the clinical differences.^45^

In contrast to patients with localized tumors, the distribution of clinical characteristics differed between patients with FP and FN metastatic aRMS. The number of patients older than 10 years, with unfavorable tumor sites, and B/BM metastases was significantly higher in the FP cohort. Age was the only factor with a significantly different distribution between patients with *PAX3* *::* vs. *PAX7* *::* *FOXO1* positive tumors. Sixty‐seven percentage of patients with *PAX3* *::* *FOXO1* positive aRMS were older than 10 years. In univariate analysis, age, B/BM metastases, fusion status and *PAX* variant were significantly associated with EFS and OS, T status with OS only; *PAX3* *::* *FOXO1* fusion remained relevant for EFS and OS in multivariate analysis, B/BM for EFS only.

Although the prognostic role of *FOXO1* fusion status is widely accepted in localized disease, it is still controversial in metastatic disease. Rudzinski et al. examined outcome depending on histology and fusion status in 178 patients with metastatic RMS (78% being diagnosed as aRMS) on COG D9802 and ARST0431.^23^ This study revealed that clinical risk factors have the most impact on outcome. Fusion status being more common in patients with higher Oberlin score but was not an independent biomarker. In contrast to our results, no significant difference was found between *PAX3* *::* *FOXO1*, *PAX7* *::* *FOXO1* and FN cases when analyzing tumors with exclusively alveolar histology.^23^ The reason for the difference in the significance of fusion status between our report and COG's may be the much lower 5‐year EFS for patients with FN aRMS (COG 29% vs. 71% in our analysis). In another COG analysis of localized and metastatic patients treated on six trials, *FOXO1* fusion emerged as the strongest prognostic variable, with no clinical factor further subdividing outcome in patients with metastatic FP tumors.^22^ However, this analysis did not distinguish between *PAX3* *::* *FOXO1* versus *PAX7* *::* *FOXO1* and FN aRMS versus eRMS. Although *PAX3* *::* *FOXO1* fusion itself has prognostic value, no molecular markers are currently available for risk stratification of FP tumors. CDK4 and MYCN amplifications have been observed in FP aRMS and appear to be associated with prognosis but future studies are needed to validate their significance.^46^ Secondary objectives of the ongoing COG ARST2031 (NCT049941329) include evaluating the impact of copy number variation, including CDK4 and MYC on outcome, which should lead to the clarification of the prognostic value of these markers. Similarly, MIR17HG may have prognostic value in *PAX7* *::* *FOXO1* positive aRMS.^47^

A limitation of our study is the fact that 22% of the tumors were not tested for fusion status. However, this is comparable to the EpSSG analysis (19%), lower than in the COG analysis for metastatic patients (37%), but higher than the COG cohort of patients with N1 positive RMS where only 7% were not tested.^23^, ^33^, ^40^

In conclusion, our study revealed many new aspects:

1. *PAX3* *::* *FOXO1* fusion status emerged as an independent predictor of poor EFS in patients with localized aRMS. On univariate analysis, clinical factors such as IRS group III and lymph node involvement were predictive of inferior EFS and OS, tumor size >5 cm, invasiveness (T2) for inferior OS.
2. In patients with metastatic disease, *PAX3* *::* *FOXO1* fusion was the independent indicator of poor EFS and OS, and B/BM metastases for poor EFS. In univariate analysis, age >10 years was additionally associated with worse EFS and OS.
3. The proportion of FN aRMS was very low (10%) compared to other published series, highlighting the difficulty of considering this group well defined and comparable between studies. They had a remarkably favorable prognosis of 5‐year EFS of 94% for localized and 71% for metastatic disease, which is better than for *PAX7* *::* *FOXO1* tumors and eRMS.
4. *PAX7* *::* *FOXO1*‐positive aRMS were associated with significantly better outcome (EFS and OS in metastatic and OS in localized disease) than *PAX3* *::* *FOXO1*‐positive aRMS (Tables 2 and 3) and with comparable outcome (EFS and OS) to eRMS in localized disease (Table S6).

Our analysis of prospectively treated patients supports the finding of Missiaglia et al. that only *PAX3* *::* *FOXO1* fusion is an independent adverse prognostic factor in patients with localized and metastatic aRMS and should replace *FOXO1* fusion in risk stratification. *PAX7* *::* *FOXO1* and FN aRMS represent subgroups with distinct prognostic relevance. Clinical factors with prognostic relevance described in this report should be considered in the risk stratification of patients with aRMS.

However, the next step should be the integration of molecular features beyond *FOXO1* fusion status into risk stratification and therapeutic decisions. Ongoing COG, CWS, and EpSSG trials and registries have included molecular characterization as accompanying studies. The results of these studies will hopefully provide the basis for new, more precise risk stratification of patients with RMS.

## AUTHOR CONTRIBUTIONS

**Ewa Koscielniak:** Conceptualization (lead); formal analysis (lead); funding acquisition (lead); methodology (lead); project administration (lead); supervision (lead); writing – original draft (lead); writing – review and editing (lead). **Sabine Stegmaier:** Conceptualization (equal); formal analysis (equal); methodology (equal); writing – original draft (equal); writing – review and editing (equal). **Gustaf Ljungman:** Formal analysis (supporting); writing – review and editing (equal). **Bernarda Kazanowska:** Investigation (equal); writing – review and editing (equal). **Felix Niggli:** Resources (equal); writing – review and editing (equal). **Ruth Ladenstein:** Resources (equal); writing – review and editing (equal). **Bernd Blank:** Data curation (equal); formal analysis (equal); software (equal); visualization (equal); writing – review and editing (equal). **Erika Hallmen:** Data curation (equal); formal analysis (equal); methodology (equal); software (equal); validation (equal); writing – review and editing (equal). **Christian Vokuhl:** Conceptualization (equal); methodology (equal); resources (equal); writing – review and editing (equal). **Claudia Blattmann:** Resources (equal); writing – review and editing (equal). **Monika Sparber‐Sauer:** Formal analysis (equal); methodology (equal); writing – review and editing (equal). **Thomas Klingebiel:** Conceptualization (equal); funding acquisition (equal); methodology (equal); writing – review and editing (equal).

## FUNDING INFORMATION

The CWS‐2002P and CWS‐IV 2002 trials were supported by grants from the German Cancer Aid Foundation. (CWS‐2002P: 50‐2721‐Tr2. CWS‐IV 2002: 50‐2695), the CWS Registry SoTiSaR from the German Children's Cancer Foundation (DKS 2009.08. 2012.04 and 2015.12) and by the Förderkreis Krebskranke Kinder Stuttgart. Germany (continuous support).

## CONFLICT OF INTEREST STATEMENT

The authors have no potential conflicts of interest related to any aspect of this analysis.

## Supporting information


Data S1.

## Data Availability

Individual participant data are not publicly available as this was not a requirement of the study and registry protocols.
