# Supplementary material for: Prognostic factors in patients with localized and metastatic alveolar rhabdomyosarcoma. A report from two studies and two registries of the Cooperative Weichteilsarkom Studiengruppe CWS
Source: Cancer Med. 2025 Jan 9;14(1):e70215. doi: 10.1002/cam4.70215 (PMC11712121; doi:10.1002/cam4.70215)
Supplement: Supplementary file 1 — Data S1. [file CAM4-14-e70215-s001.docx]

**Supporting information**

**Methods.**

1. Staging system

2. Chemotherapy description

3. Genes of the panel

**Supporting Tables**

Table S1. Risk stratification for patients with localized rhabdomyosarcoma

Table S2. Distribution of patients and tumor related variables in patients with FP vs. FN localized aRMS.

Table S3. Distribution of patients and tumor related variables in patients with FP vs. FN metastatic aRMS

Table S4. Distribution of patients and tumor related variables in patients with *PAX3::FOXO1* vs. *PAX7::FOXO1* bearing, localized aRMS.

Table S5. Distribution of patients and tumor related variables in patients with *PAX3::FOXO1* vs. *PAX7::FOXO1* bearing, metastatic aRMS.

Table S6**.** Comparison of EFS and OS of patients with *PAX3::FOXO1*, *PAX7::FOXO1* positive tumors and FN aRMS with patients with eRMS

**Supporting Figures**

Figure S1. CWS-2002P Treatment plan

Figure S2. CWS-Guidance Treatment plan

**Legends**

**References**

**Methods**

1. Staging system

Staging procedures included magnetic resonance imaging (MRI) of the primary site and if indicated computed tomography (CT) and ultrasound. Metastatic disease was assessed by chest CT, optionally whole body MRI in the latest study, cerebral MRI or CT, technetium bone scan, bone marrow aspiration or trephine biopsy. The TNM classification was applied to differentiate pre-treatment and postsurgical stages^1^. The clinical grouping system (IRS I, II, III) adapted from the Intergroup Rhabdomyosarcoma Study (IRS) post-surgical grouping system was used to categorize patients according to primary surgery^2^. Resection was classified as R0 (free resections margins), R1 (microscopically incomplete), or R2 (macroscopically incomplete).

1. Chemotherapy description
   Patients enrolled in SoTiSaR were treated according to the CWS-Guidance treatment recommendations, which were developed based on the CWS-2002P study in collaboration with the International Society for Pediatric Oncology-Malignant Mesenchymal Tumor Group (MMT SIOP) and Associazione Italiana di Ematologia e Oncologia Pediatrica –Soft Tissue Sarcoma Committee and for patients with localized tumors are identical to the standard therapy arm of the EpSSG RMS 2005 trial^3-5^.

Patients with localized aRMS enrolled on CWS-2002P and SoTiSaR were assigned to HR and Very High risk groups and treated with vincristine, ifosfamide, dactinomycin and doxorubicin as combinations of three (IVA) or four drugs (VAIA), Figures S1 and S2. In the CWS-2002P study maintenance chemotherapy with cyclophosphamide and vinblastine (CYC/VBL) was recommended as an option at the end of the multimodal intensive therapy Patients with metastatic aRMS enrolled in CWS-DOK IV 2004 and SoTiSaR were treated with six drugs (additionally etoposide, carboplatin and epirubicin instead of doxorubicin, CEVAIE) that were used in the CWS studies for patients with metastatic tumors based on the results of the European Intergroup Studies MMT4-89 and -91^6^. CWS-IV-2002, a Phase II window study, evaluated the efficacy of two courses of topotecan and carboplatin (TC) prior to treatment with CEVAIE. The windows trial did not result in improved outcome^7^. Patients with metastatic tumors who achieved complete remission at the end of the standard therapy were offered oral maintenance therapy O-TIE consisted of 10 day courses of trofosfamide (2x75mg/m^2^/d) and etoposide (2x25 mg/m^2^/d) (TE) alternating with trofosfamide (2x75mg/m^2^/d) and idarubicine (1x5mg on day one) based on the results of the HD CWS-96 study^8^.

1. Genes of the panel: ALK, BCOR, BRAF, CAMTA1, CCNB3, DNAJB1, EPC1, ERG, EWSR1, FGFR1, FGFR2, FGFR3, FOXO1, FUS, GLI1, GLIS1, GLIS3, HMGA2, JAZF1, MAML2, MEAF6, MET, MKL2, MYB, MYBL, NCOA2, NFIB, NTRK1, NTRK2, NTRK3, NUTM1, NUTM2A, NUTM2B, NUTM2E, PAX3, PAX8, PDGFB, PDGFRA, PLAG1, PPARG, PRKACA, PRKCA, RAF1, RELA, RET, ROS1, SS18, STAT6, TAF15, TCF12, TFE3, TFEB, TFG, TMPRSS2, USP6, VGLL2, YAP1, YWHAE

**Supporting Tables and Figures**

**Table S1.** Risk stratification for patients with localized rhabdomyosarcoma CWS 2002P and SoTiSaR

| Risk Group | Pathology | IRS Group | Site | LN Stage | Size&Age | T Status |  |  |
| --- | --- | --- | --- | --- | --- | --- | --- | --- |
| LR | eRMS | I | Any | N0 | Favorable |  |  |  |
| SR | eRMS | I | Any | N0 | Unfavorable | - |  |  |
|  | eRMS | II, III | Favorable | N0 | Any | - |  |  |
|  | eRMS | II, III | Unfavorable | N0 | Favorable | - |  |  |
| HR | eRMS | II, III | Unfavorable | N0 | Unfavorable | - |  |  |
|  | eRMS | II, III | Any | N1 | Any | - |  |  |
|  | aRMS | I, II, III | Any | N0 | Any | - |  |  |
| VHR | aRMS | II, III | Any | N1 | Any | - |  |  |

Abbreviations and/or definitions: IRS Group - postsurgical stage: LR low risk, SR standard risk, HR high risk, VHR very high risk; Pathology: eRMS – all embryonal RMS spindle cell and botryoid, aRMS–all alveolar RMS (including solid-alveolar variant); Site – primary tumor site Favorable: Orbit (ORB), Genitourinary Non-Bladder&Prostate GU-NBP (i.e. paratesticular, vagina/uterus), Head&Neck Non-Paramenigeal (HN-NPM), Site – primary tumor site Unfavorable: Head&Neck Paramenigeal HN-PM, Genitourinary Bladder&Prostate (GU-BP), Extremities (EXT), “Other Site” (OTH); LN Stage – regional lymph node status; Size&Age Favorable: ≤ 5 cm and ≤10 years, Size&Age Unfavorable: > 10 years and/or > 5 cm

**Table S2.** Distribution of patients and tumor related variables in patients with FP vs. FN localized aRMS

| **Variable** | **Total** | **Total%** | **FN** | **FN %** | **FP** | **FP %** | **p Fisher** |
| --- | --- | --- | --- | --- | --- | --- | --- |
|  | **163** |  | **19** |  | **144** |  |  |
| **Gender** |  |  |  |  |  |  | 1.0 |
| Male | 83 | 50.9 | 10 | 52.6 | 73 | 50.7 |  |
| female | 79 | 48.5 | 9 | 47.4 | 70 | 48.6 |  |
| missing data | 1 | 0.6 | 0 | 0.0 | 1 | 0.7 |  |
| **Age** |  |  |  |  |  |  | 0.32 |
| ≤10 yrs | 99 | 60.7 | 14 | 73.7 | 85 | 59.0 |  |
| >10 yrs | 64 | 39.3 | 5 | 26.3 | 59 | 41.0 |  |
| missing data | 0 | 0.0 | 0 | 0.0 | 0 | 0.0 |  |
| **Tumor Size** |  |  |  |  |  |  | 1.0 |
| ≤5cm | 77 | 47.2 | 9 | 47.4 | 68 | 47.2 |  |
| >5cm | 75 | 46.0 | 9 | 47.4 | 66 | 45.8 |  |
| missing data | 11 | 6.7 | 1 | 5.3 | 10 | 6.9 |  |
| **Tumor Site Risk group** |  |  |  |  |  |  | 1.0 |
| Favorable ( ORB, GU-nBP, HN-nPM)* | 39 | 23.9 | 4 | 21.1 | 35 | 24.3 |  |
| Unfavorable (EXT, HN-PM, GU-BP, Other)** | 124 | 76.1 | 15 | 78.9 | 109 | 75.7 |  |
| **Tumor Site** |  |  |  |  |  |  | 0.57 |
| EXT | 51 | 31.3 | 4 | 21.1 | 47 | 32.6 |  |
| HN-nPM | 31 | 19.0 | 3 | 15.8 | 28 | 19.4 |  |
| HN-PM | 43 | 26.4 | 6 | 31.6 | 37 | 25.7 |  |
| ORBITA | 4 | 2.5 | 0 | 0.0 | 4 | 2.8 |  |
| GU-BP | 4 | 2.5 | 1 | 5.3 | 3 | 2.1 |  |
| GU-nBP | 4 | 2.5 | 1 | 5.3 | 3 | 2.1 |  |
| OTHER | 26 | 16.0 | 4 | 21.1 | 22 | 15.3 |  |
| **T-Status** |  |  |  |  |  |  | 0.80 |
| T1 | 73 | 44.8 | 9 | 47.4 | 64 | 44.4 |  |
| T2 | 74 | 45.4 | 8 | 42.1 | 66 | 45.8 |  |
| TX | 16 | 9.8 | 2 | 10.5 | 14 | 9.7 |  |
| **N-Status** |  |  |  |  |  |  | 0.32 |
| N0 | 95 | 58.3 | 14 | 73.7 | 81 | 56.3 |  |
| N1 | 59 | 36.2 | 5 | 26.3 | 54 | 37.5 |  |
| NX | 9 | 5.5 | 0 | 0.0 | 9 | 6.2 |  |

*ORB- Orbita, GU-nBP- Genitourinary-non Bladder/Prostate, HN-nPM - Head/Neck-non Parameningeal,

**EXT- Extremity, HN-PM- Head/Neck-Parameningeal, GU-BP- Genitourinary-Bladder/Prostate

**Table S3.** Distribution of patients and tumor related variables in patients with FP vs. FN. Metastatic aRMS

|  | **Total** | **Total%** | **FN** | **FN%** | **FP** | **FP%** | **p Fisher** |
| --- | --- | --- | --- | --- | --- | --- | --- |
|  | **205** |  | **19** |  | **186** |  |  |
| **Gender** |  |  |  |  |  |  | 1.0 |
| male | 100 | 48.8 | 9 | 47.4 | 91 | 48.9 |  |
| female | 104 | 50.7 | 10 | 52.6 | 94 | 50.5 |  |
| missing data | 1 | 0.5 | 0 | 0.0 | 1 | 0.5 |  |
| **Age** |  |  |  |  |  |  | 0.002 |
| ≤10 yrs | 79 | 38.5 | 14 | 73.7 | 65 | 34.9 |  |
| >10 yrs | 125 | 61.0 | 5 | 26.3 | 120 | 64.5 |  |
| missing data | 1 | 0.5 |  | 0.0 | 1 | 0.5 |  |
| Tumor Size |  |  |  |  |  |  | 1.0 |
| ≤5cm | 48 | 23.4 | 5 | 26.3 | 43 | 23.1 |  |
| >5cm | 132 | 64.4 | 14 | 73.7 | 118 | 63.4 |  |
| missing data | 25 | 12.2 | 0 | 0.0 | 25 | 13.4 |  |
| **Tumor Site**  **Risk group** |  |  |  |  |  |  | 0.01 |
| Favorable*  (ORB, GU-nBP, HN-nPM) | 6 | 2.9 | 3 | 15.8 | 3 | 1.6 |  |
| Unfavorable**  (EXT, HN-PM, GU-BP, Other)** | 194 | 94.6 | 16 | 84.2 | 178 | 95.7 |  |
| missing data | 5 | 2.4 | 0 | 0.0 | 5 | 2.7 |  |
| **Tumor Site** |  |  |  |  |  |  | 0.0001 |
| EXT | 98 | 47.8 | 3 | 15.8 | 95 | 51.1 |  |
| HN-nPM | 2 | 1.0 | 1 | 5.3 | 1 | 0.5 |  |
| HN-PM | 23 | 11.2 | 7 | 36.8 | 16 | 8.6 |  |
| ORBITA | 0 | 0.0 | 0 | 0.0 | 0 | 0.0 |  |
| UG_BP | 13 | 6.3 | 1 | 5.3 | 12 | 6.5 |  |
| UG-nBP | 4 | 2.0 | 2 | 10.5 | 2 | 1.1 |  |
| OTHER | 60 | 29.3 | 5 | 26.3 | 55 | 29.6 |  |
| missing data | 5 | 2.4 | 0 | 0.0 | 5 | 2.7 |  |
| **T-Status** |  |  |  |  |  |  | 1.0 |
| T1 | 24 | 11.7 | 2 | 10.5 | 22 | 11.8 |  |
| T2 | 165 | 80.5 | 16 | 84.2 | 149 | 80.1 |  |
| TX | 16 | 7.8 | 1 | 5.3 | 15 | 8.1 |  |
| **Bone/Bone marrow metastases** |  |  |  |  |  |  | 0.004 |
| yes | 139 | 67.8 | 7 | 36.8 | 132 | 0.71 |  |
| no | 66 | 32.2 | 12 | 63.2 | 54 | 0.29 |  |

*ORB- Orbita, GU-nBP- Genitourinary-non Bladder/Prostate, HN-nPM - Head/Neck-non Parameningeal,

** EXT- Extremity, HN-PM- Head/Neck-Parameningeal, GU-BP- Genitourinary-Bladder/Prostate, Other

**Table S4**. Distribution of patients and tumor related variables in patients with *PAX3::OXO1* vs. *PAX7::FOXO1* bearing, localized aRMS.

| **Variable** | **PAX3** | **%** | **PAX 7** | **%** | **p Fisher** |
| --- | --- | --- | --- | --- | --- |
|  | **93** |  | **25** |  |  |
| **Gender** |  |  |  |  | 0.37 |
| Male | 48 | 51.6 | 10 | 40.0 |  |
| female | 44 | 47.3 | 15 | 60.0 |  |
| missing data | 1 | 1.1 | 0 | 0 |  |
| **Age** |  |  |  |  | 0.10 |
| ≤10 yrs | 53 | 57.0 | 19 | 76.0 |  |
| >10 yrs | 40 | 43.0 | 6 | 24.0 |  |
| missing data | 0 | 0 | 0 | 0 |  |
| **Tumor Size** |  |  |  |  | 1.0 |
| ≤5cm | 46 | 49.5 | 13 | 52.0 |  |
| >5cm | 39 | 42.0 | 12 | 48.0 |  |
| missing data | 8 | 8.5 | 0 | 0 |  |
| **Tumor Site Risk group** |  |  |  |  | 0.80 |
| Favorable ( ORB, GU-nBP, HN-nPM)* | 24 | 25.8 | 7 | 28.0 |  |
| Unfavorable (EXT, HN-PM, GU-BP, Other)** | 69 | 74.2 | 18 | 72.0 |  |
| missing data | 0 | 0 | 0 | 0 |  |
| **T-Status** |  |  |  |  | 0.04 |
| T1 | 34 | 36.6 | 16 | 64.0 |  |
| T2 | 49 | 52.7 | 7 | 28.0 |  |
| misval | 10 | 10.7 | 2 | 8.0 |  |
| **N-Status** |  |  |  |  | 0.49 |
| N0 | 51 | 54.8 | 17 | 68.0 |  |
| N1 | 38 | 40.9 | 7 | 28.0 |  |
| misval | 4 | 4.3 | 1 | 4.0 |  |

*ORB- Orbita, GU-nBP- Genitourinary-non Bladder/Prostate, HN-nPM - Head/Neck-non Parameningeal,

** EXT- Extremity, HN-PM- Head/Neck-Parameningeal, GU-BP- Genitourinary-Bladder/Prostate, Other

**Table S5**. Distribution of patients and tumor related variables in patients with *PAX3::FOXO1* vs. *PAX7::FOXO1* bearing, metastatic aRMS.

| **Variable** | **PAX3** | **%** | **PAX 7** | **%** | **p Fisher** |
| --- | --- | --- | --- | --- | --- |
|  | **138** |  | **24** |  |  |
| **Gender** |  |  |  |  | 0.11 |
| Male | 71 | 44.3 | 10 | 41.7 |  |
| female | 67 | 55.7 | 13 | 54.2 |  |
| missing data | 0 | 0 | 1 | 4.1 |  |
| **Age** |  |  |  |  | 0.002 |
| ≤10 yrs | 44 | 31.9 | 16 | 67.0 |  |
| >10 yrs | 94 | 68.1 | 8 | 33.0 |  |
| **Tumor Size** |  |  |  |  | 0.65 |
| ≤5cm | 33 | 23.9 | 4 | 17.0 |  |
| >5cm | 87 | 63.0 | 16 | 66.0 |  |
| missing data | 18 | 13.1 | 4 | 17.0 |  |
| **Tumor Site Risk group** |  |  |  |  | 0.68 |
| Favorable ( ORB, GU-nBP, HN-nPM)* | 2 | 1.5 | 0 | 0 |  |
| Unfavorable (EXT, HN-PM, GU-BP, Other)** | 134 | 97.0 | 23 | 87.5 |  |
| missing data | 2 | 1.5 | 1 | 12.5 |  |
| **T-Status** |  |  |  |  | 0.71 |
| T1 | 14 | 10.1 | 3 | 12.5 |  |
| T2 | 110 | 79.7 | 20 | 83.3 |  |
| Missing data | 14 | 19.2 | 1 |  |  |
| **N-Status** |  |  |  |  | 0.26 |
| N0 | 32 | 23.2 | 2 | 8.3 |  |
| N1 | 83 | 60.1 | 17 | 70.8 |  |
| missing data | 23 | 16.7 | 5 | 20.9 |  |
| **B/BM Metastases** |  |  |  |  | 0.22 |
| Yes | 101 | 73.2 | 15 | 62.5 |  |
| No | 37 | 26.8 | 9 | 37.5 |  |

*ORB- Orbita, GU-nBP- Genitourinary-non Bladder/Prostate, HN-nPM - Head/Neck-non Parameningeal,

** EXT- Extremity, HN-PM- Head/Neck-Parameningeal, GU-BP- Genitourinary-Bladder/Prostate, Other

**Table S6.** Comparison of 5-year EFS and OS of patients with *PAX3::FOXO1*, *PAX7::FOXO1* positive and FN aRMS with patients with eRMS

| **Localized** |  |  |  |  |  |
| --- | --- | --- | --- | --- | --- |
| **Fusion Status*** | **n** | **5 year EFS** | **P logrank vs. eRMS** | **5 year OS** | **P logrank vs. eRMS** |
| PAX3 | 93 | 42.0 (32.8-53.8) | 1.3e-12 | 50.5 (40.4-63.1) | 4.0e-12 |
| PAX7 | 25 | 60.4 (43.1-84.7) | 0.09 | 80.9 (65.6-99.7) | 0.76 |
| FN | 19 | 93.7 (82.6-100.0) | 0.08 | 93.7 (82.6-100.0) | 0.26 |
| eRMS | 811 | 74.5 (71.3-77.8) |  | 83.6 (80.8-86.6) |  |
| **Metastatic** |  |  |  |  |  |
| **Fusion status*** | **n** | **5 year EFS** | **P logrank vs. eRMS** | **5 year OS** | **P logrank vs.eRMS** |
| PAX3 | 138 | 7.7 (4.2-14.0) | 9.2 e -15 | 9.8 (5.6-17.1) | 1.0e -15 |
| PAX7 | 24 | 17.2 (6.6-44.7) | 0.01 | 28.3 (14.1-56.9) | 0.01 |
| FN | 19 | 71.0 (52.3-96.4) | 0.07 | 70.1 (51.0-96.1) | 0.19 |
| eRMS | 158 | 45.0 (37.4-54.4) |  | 55.8 (47.9-64.9) |  |

*We did not include patients with FP tumors but without a known PAX partner in this analysis.

**Figures S1.**


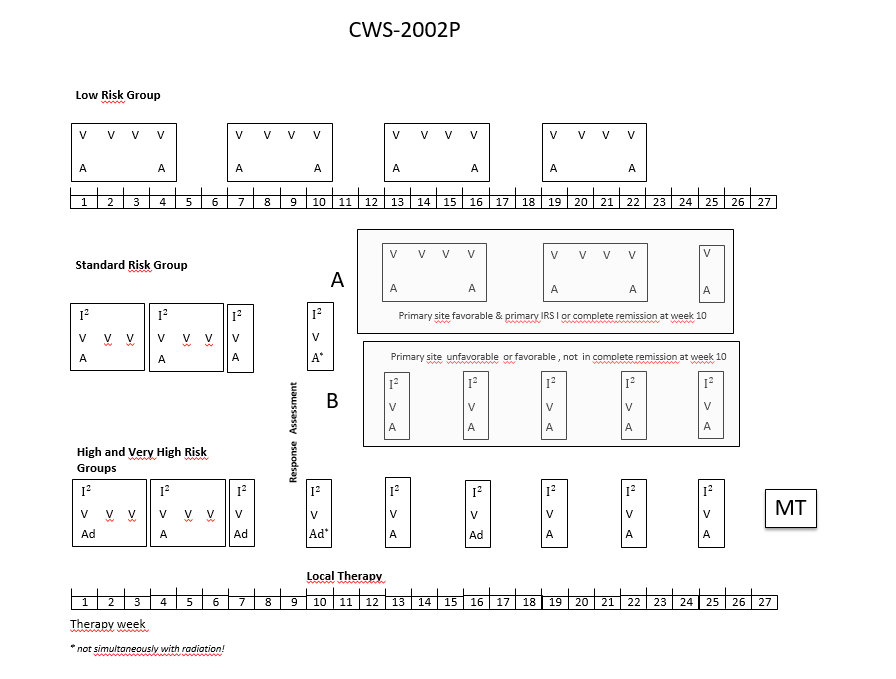


**Figure S2.**


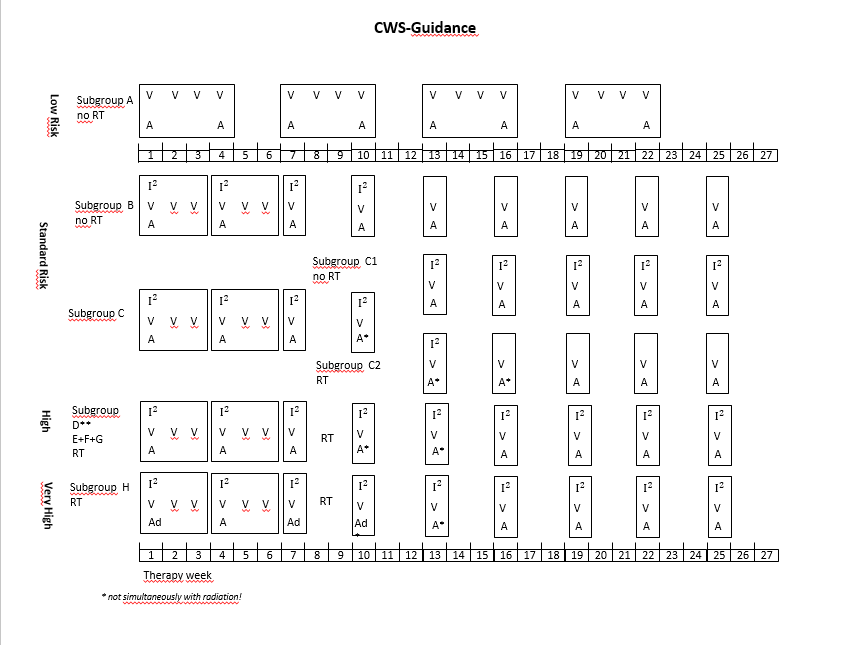


**Legends**

**Figure S1. CWS-2002P Treatment plan**.

V Vincristine 1.5mg/m²/d; maximum, 2 mg/day, I^2^Ifosfamide 3 g/m²/d, on two consecutive days, A dactinomycin 1.5 mg/m²/d maximum, 1.5 mg/day, Ad Doxorubicin 40mg/m²/day, on two consecutive days , MT, maintenance therapy, (optionally for patients who achieved CR at the end of multimodal standard therapy): Cyclophosphamide/Vinblastine: seven 3-weeks cycles of intravenous vinblastine 3mg/m² on days 1, 8 and 15 and oral cyclophosphamide 2 x 25mg/m²/day days 1-21, with one week pause between the cycles. The dosage of MT should be adapted to the leukocyte count, which should not be below 1500/µl.

**Figure S2. CWS-Guidance Treatment plan**

V Vincristine 1.5mg/m²/d; maximum, 2 mg/day, I^2^Ifosfamide 3 g/m²/d, on two consecutive days, A dactinomycin 1.5 mg/m²/d maximum, 1.5 mg/day, Ad Doxorubicin 40mg/m²/day, on two consecutive days

Subgroup C1 Only patients with favorable size and age and secondary R0- resetion

Subgroup C2 All other patients

**Subgroup D was formally part of the standard risk group but the therapy was the same as in the high-risk group

**References**

1. Rodary C, Flamant F, Donaldson SS. An attempt to use a common staging system in rhabdomyosarcoma: a report of an international workshop initiated by the International Society of Pediatric Oncology (SIOP). *Medical and pediatric oncology.* 1989;17(3):210-215.

2. Maurer HM, Beltangady M, Gehan EA, et al. The Intergroup Rhabdomyosarcoma Study-I. A final report. *Cancer.* 1988;61(2):209-220.

3. Koscielniak E, Blank B, Vokuhl C, et al. Long-Term Clinical Outcome and Prognostic Factors of Children and Adolescents with Localized Rhabdomyosarcoma Treated on the CWS-2002P Protocol. *Cancers.* 2022;14(4):899.

4. Koscielniak E, Timmermann B, Munter M, et al. Which Patients With Rhabdomyosarcoma Need Radiotherapy? Analysis of the Radiotherapy Strategies of the CWS-96 and CWS-2002P Studies and SoTiSaR Registry. *Journal of clinical oncology : official journal of the American Society of Clinical Oncology.* 2023:JCO2202673.

5. Bisogno G, Minard-Colin V, Zanetti I, et al. Nonmetastatic Rhabdomyosarcoma in Children and Adolescents: Overall Results of the European Pediatric Soft Tissue Sarcoma Study Group RMS2005 Study. *Journal of clinical oncology : official journal of the American Society of Clinical Oncology.* 2023;41(13):2342-2349.

6. Carli M, Colombatti R, Oberlin O, et al. European intergroup studies (MMT4-89 and MMT4-91) on childhood metastatic rhabdomyosarcoma: final results and analysis of prognostic factors. *Journal of clinical oncology : official journal of the American Society of Clinical Oncology.* 2004;22(23):4787-4794.

7. Bochennek K, Dantonello T, Koscielniak E, et al. Response of children with stage IV soft tissue sarcoma to topotecan and carboplatin: a phase II window trial of the cooperative soft tissue sarcoma group. *Klinische Padiatrie.* 2013;225(6):309-314.

8. Klingebiel T, Boos J, Beske F, et al. Treatment of children with metastatic soft tissue sarcoma with oral maintenance compared to high dose chemotherapy: report of the HD CWS-96 trial. *Pediatric blood & cancer.* 2008;50(4):739-745.
